# Green treasures: Investigating the biodiversity potential of equine yards through the presence and quality of landscape features in the Netherlands

**DOI:** 10.1371/journal.pone.0301168

**Published:** 2024-04-11

**Authors:** Inga A. Wolframm, Lara Heric, Andrew M. Allen

**Affiliations:** Applied Research Centre, Van Hall Larenstein University of Applied Sciences, Velp, Gelderland, Netherlands; Massey University, NEW ZEALAND

## Abstract

At a time of mounting ecological crises and biodiversity loss, there is an urgent need for nature-based solutions. Equestrian properties cover a considerable proportion of the European rural and peri-urban landscape and provide much potential for integrating ecosystem services, such as the inclusion of small landscape features. The aim of this study was to investigate the presence and quality of landscape features (LF) to help determine how the equine sector can contribute to the agro-ecological transition. Using a citizen science approach, 87 commercial and 420 private yard owners reported the type, frequency and geometric dimension of LFs and additional biodiversity enhancing features. A hierarchical multivariate regression was used to determine how equine property characteristics explain variation in the Percentage Property Coverage (PPC) of LFs. The model explained 47% of the variation of PPC. The variables that explained significant variation in PPC included Yard size, Number of LFs, Tree rows, Fruit orchard, Wild hedges, Flowering strips, Buffer strips, Embankments and Cluttered corners. Commercial yards are significantly larger with significantly more horses and on average only 9% (±13.87%) of the property was covered by LFs whilst private yards had significantly more coverage of LFs with on average 12% (±14.77%). These findings highlight the substantial yet untapped potential of equine yards in fostering biodiversity, suggesting that the equine sector could play an important role in the agro-ecological transition. To encourage more biodiverse-inclusive yard designs, tailored strategies should consider the diverse factors influencing equine yard design, including existing knowledge, client demands, financial considerations, and equine health and welfare.

## Introduction

There has been an unprecedented increase in extreme and adverse weather events [1], a staggering loss of biodiversity amounting to the Sixth Mass Extinction [2], a decline in quality of water [3] and air [4], an increase in world hunger [5]. The numerous direct and indirect impacts of human activities would likely result in scientists officially announcing the start of a new epoch (the Anthropocene era) to denote the impact human beings have had on the earth’s ecosystems [6]. As a result, there is an urgent need for innovative solutions that help mitigate the effects of human activities such as land use and climate change, while also assisting society to adapt to current and future situations.

Nature itself is increasingly being viewed as one of the most important assets to mitigate the effects of climate change [1]. The concept of ecosystem services as a means to raise awareness to the benefits of the ecological environment to human society was devised in the late 1970s and early 1980s [7, 8]. However, the increasing threat of climate change has accelerated the need to understand how anthropogenic management of landscapes might affect the functioning of the natural environment [9].

Small landscape features (LF) have long since been considered important elements of such ecosystem services. Small LFs are defined as ‘small fragments of non-productive natural or semi-natural vegetation in agricultural landscapes which provide ecosystem services and support for biodiversity [10]. Examples include hedges, ponds, individual trees, trees in lines or groups, field margins and stone or earth walls [10, 11]. LFs have numerous functional uses, such as the provision of windbreaks and shelter for livestock, regulation of, and protection from, soil erosion, management of water quantity and quality, enhancement of air quality, CO2 sequestration, as well as the maintenance and protection of biodiversity [9, 11–16].

In the EU, around 45% of land cover is occupied by agriculture [10], meaning that many of the efforts to protect, maintain, restore and create LFs have been integrated into the Common Agricultural Policy (CAP). This was meant to be achieved by a number of legal requirements covered in several regulations, rural development programmes and greening measures, which included the creation and retention of Ecological Focus Areas (EFA) [11]. In order to acknowledge agricultural, ecological and cultural differences, EU Member States were able to determine which types of areas to include in their national EFAs, in order to convert at least 10% of agricultural area into high-diversity landscape features by 2030 [17]. However, despite the undeniable significance of LFs in preserving biodiversity and ecosystems, member states exhibited hesitancy in incorporating landscape features as EFAs, favouring options such as green cover, catch crops, and fallow fields instead [10, 11]. Consequently, LFs currently account for less than 2% of the total EFA area at the EU level [10]. It seems clear that more concerted efforts are needed to accelerate the integration of LFs across Europe [10, 18].

Up until now, the equine sector has largely been overlooked as a player in the current agro-ecological transition [19, 20]. In part, this may be due to differences in the legal definition of horses in different countries, which depend on their definition of purpose, i.e. leisure, companionship, sport, therapy, agriculture, forestry and more [21]. However, regardless of their legal designation, the keeping and management of horses may offer considerable environmental benefits. Recent research has identified five ‘Green assets’ [20], including Equine grazing, domestic biodiversity, land use, tourism and equine work. As highlighted by Rzekęć et al., [20], due to their physiological and ethological needs, horse keeping is generally associated with considerable land use. In fact, across the EU, the land coverage associated with horses is estimated at six million hectares of primarily permanent grassland [22]. Not surprisingly, therefore, the limited research efforts that have been undertaken, have largely focused on the equine industry in terms of grassland management [19, 20, 23–25]. As opposed to grassland in an agricultural setting, equine grassland is largely non-productive and may provide ecosystem services more commonly associated with LFs. Furthermore, it stands to reason that equine properties offer considerable potential regarding the integration of different types of LFs. After all, much of the traditional uses of LFs, i.e., to offer shelter from the elements, provide additional feed sources, or be used as means of enclosure, or for the separation of different fields, continue to be appropriate ecosystem services to horse owners in their efforts to provide horses with management systems that safeguard their health and welfare [24, 26–28].

The Netherlands is considered one of the most prolific equestrian nations worldwide. With approximately 450,000 riders and 500,000 horses, equestrianism is considered one of the most popular sports [29]. Previous research suggested that these horses are kept at approximately 81,000 locations [30], of which 10,000 are businesses such as livery yards, riding schools, as well as breeding and training stables. The rest are likely to be private locations where horses are kept either at home, or on privately rented land. Considering that the Netherlands is confronted with a combination of spatial restrictions, a prominent agricultural sector and stringent EU requirements regarding the protection and restoration of biodiversity resources [31, 32], the equine sector may likely play an important role in the preservation and improvement of land-based biodiversity levels through the integration of LFs.

In order to determine the biodiversity potential of equine land use it is important to gain an overview of the prevalence, types and area coverage of LFs across different types of equine yards. Increasingly, studies aimed at collecting environmental data with extensive geographical coverage rely on the involvement of volunteers to gather data [33–35]. Considering the logistical, organisational, and financial challenges in conducting a traditional data collection study across the different types of equine yards, such a citizen science approach was considered an effective way of gathering data. Therefore, the aim of the current study was to gauge the presence, frequency and geometric properties of LFs on private and commercial equine yards, including in relation to property size, across the Netherlands, drawing on the active participation of yard owners.

## Method

### Survey design

In order to engage as many yard owners as possible across the Netherlands, a cross-sectional study design was used, targeting Dutch private horse owners who kept their horses at home, as well as commercial equine yard owners who managed their own land. The survey included a total of 152 questions in Dutch and was designed in the online survey tool Survey Monkey.

In the first part, participants were asked to provide information on whether the yard was privately or commercially owned, the general geographic location (i.e. region), size of the property, designation (i.e. type of agricultural activities allowed); soil type and number of horses kept on the property. For the second part, participants were asked to provide information on the presence and, where applicable, size of landscape features (LFs) present, as well as other biodiversity enhancing attributes (Table 1). The LFs included in the survey were determined according to guidelines from Stichting Part-Ner, Nature Certification, and cross-checked with the EU Functional Landscape Feature classes according to Czúcz, Baruth, Terres, et al. [10] (Table 1).

**Table 1:** Content of survey questions regarding LFs and biodiversity features (with *denoting the elements that were included in the calculation of overall Percentage Property Coverage (PPC).

Type of landscape and biodiversity features	Qualifying criteria	
	Number	Size
**Solitary tree **	Number present	Average circumference (cm) measured 1.3 m from the ground
**Monumental tree **	Number present	Average circumference (cm) measured 1.3 m from the ground
**Pollard tree **	Number present	Average circumference (cm) measured 1.3 m from the ground
Tree lane*	Number present	Average length and width (m)
Tree row*	Number present	Average length and width (m)
Fruit orchard*	Number present	Average length and width (m)
Hedgerow*	Number present	Average length and width (m)
Trimmed hedge*	Number present	Average length and width (m)
Woody strip*	Number present	Average length and width (m)
Forage wall*	Number present	Average length and width (m)
Flowering strip*	Number present	Average length and width (m)
Embankment*	Number present	Average length and width (m)
Buffer strip*	Number present	Average size in m^2^
**Monocultural grassland **	n/a	Average size in m^2^
**Herbaceous grassland **	n/a	Average size in m^2^
Pond*	Number present	Average size in m^2^
Permanent woodpile*	Number present	Average size in m^2^
Cluttered corner*	Number present	Average size in m^2^
**Birdhouse **	Number present	n/a
**Bat box **	Number present	n/a
**Insect hotel **	Number present	n/a
**Proven nesting site **	Number present	Average size in m^2^

### Participants

Participants were recruited via social media by posting the link to the survey on social media channels from Van Hall Larenstein (VHL), relevant equestrian partners and researchers’ personal accounts. Further distribution relied on a snowball effect for maximum impact. Participation was voluntary and participants were asked to actively consent to take part in the study. No personal information was included as part of the data collection, but participants could leave their email address if they were interested in participating in future research relating to biodiversity in equine yards. The study was conducted in line with the Dutch Code of Conduct for Research Integrity.

### Data classification

To determine whether landscape features from the current survey would qualify as LFs in terms of functional size, and could thus contribute to biodiversity, all landscape features were classified using the geometric specifications of the EU Functional Landscape Feature Classes (FLFC) as set out by Czúcz, Baruth, Terres, et al. [10] (see Table 2). Whenever LFs did not comply with these specifications in terms of functional size, i.e., because they were either too large, or too small, and therefore did not qualify as a LF, they were considered as not contributing towards biodiversity and were thus not included in any further analyses involving LFs.

**Table 2:** The proposed EU Functional Landscape Feature Classes cross-referenced to the LF types commonly recognized in policy documents and their geometric specifications according to Czúcz, Baruth, Terres, et al. [10], and the concurrent classification of current survey items.

EU Functional LF (FFLF) class	Examples for commonly recognized subtypes according to Czúcz, Baruth, Terres, et al. [10]	The proposed functional landscape feature classes with geometric specifications	Classification of current survey items
**Woody features **	Isolated trees, Tree lines and avenues, hedges, woody strips, trees in group, field coppices and riparian woody vegetation	width > = 1 m AND (width < = 20 m OR area < = 0.5 ha)	Isolated trees (e.g. solitary, monumental and pollard trees), tree lanes (i.e. avenues), tree rows (i.e. lines), fruit orchards, wild hedges, trimmed hedges, woody strip, forage wall, permanent woodpile
**Grassy features **	Grassy strips, field margins, embankments, buffer strips, grassed ’thalweg’	width > = 1 m AND (width < = 20 m OR area < = 0.5 ha)	Flowering strips, embankment, buffer strip
**Wet features **	Inland channels of fresh water, standing small water bodies such as natural or man-made ponds, ditches.	width > = 1 m AND (width < = 20 m OR area < = 0.5 ha)	Pond
**Stony features **	Dry stone walls, terrace elements, rock outcrops, natural or artificial stacks of stone.	(width OR height) > = 1m AND (width < = 20 m OR area < = 0.5 ha)	Cluttered corner

A number of features not listed in the EU’s proposed FLFC, were included in the current survey. Where these features functions could be argued to resemble commonly classified LFs, they were considered as such, e.g. solitary, monumental and pollard trees were considered isolated trees and the function of permanent woodpiles and cluttered corners may be considered similar to rock piles. Additionally, features including monocultural and herbaceous grassland, birdhouses, bat boxes, insect hotels and proven nesting sites are not considered in the EU FLFC. However, they can have potential utility in contributing towards biodiversity [36–38]. Therefore, it would be beneficial to investigate their presence on farms to help gauge potential importance and determine whether further investigation on these types of landscape feature is warranted.

### Data analysis

Once the survey was closed, all responses were downloaded and transferred to IBM SPSS for Social Scientists 28.0. There were a total of 1149 responses to the survey, with 529 completions. Only these responses were considered for further analysis.

Descriptive statistics were conducted to describe the characteristics of the yards, outlining type of yard, i.e. ownership, yard size, number of horses, soil type, number of LFs and size of LFs. Descriptive statistics were also calculated for all LFs, whereby the geometric mean, median and mode was calculated for all LFs that could be measured.

Percentage Property Coverage (PPC) with LFs was calculated using the following formula:

$$PPC=\frac{TPA}{TLFC}$$ (1)

Total property area (TPA) was defined as the total area of the equestrian yard and total landscape feature coverage (TLFC) was composed of the sum of the surface of the two-dimensional (length x width) woody, grassy, wet and stony features as indicated in Table 1 with an * (i.e. all woody elements, excluding vertical LFs of solitary, monumental and pollard tree; all grassy features excluding monocultural and herbal grassland, all wet features, and all stony features). Data were visually inspected for unrealistic outliers (e.g. 100 hedgerows at a property of 2 ha) and a total of 22 individual responses were removed due to (suspected) erroneous data (e.g. coverage of LFs exceeding the size of the property).

All variables were tested for normality using the Kolmogorov-Smirnov Test which revealed non-normal distribution across all variables. A Mann-Whitney U test was conducted to determine significant differences between commercial and private yards for Yard size, Number of horses, Number of LFs and PPC.

univariable linear regression analysis was conducted initially to test the unique contribution of twenty-one (N = 21) predictor variables on PPC, including five (N = 5) “yard descriptors” (Type of ownership, Yard size, Number of horses, Soil type and Number of LFs and fifteen (N = 15) two dimensional LFs, namely Tree lanes, Tree rows, Fruit orchard, Wild hedges, Trimmed hedges, Woody strips, Forage walls, Flowering strips, Embankments, Buffer strips, Ponds, Permanent woodpiles, Cluttered corners, and Monocultural grassland and Herbaceous grassland. Even though the variables Monocultural grassland and Herbaceous grassland were not included in the calculation of PPC, they might be argued to be of importance to the total level of biodiversity, and were therefore included in the regression analysis.

Variables were selected for inclusion in the hierarchical regression model based on their statistical significance in univariable regression analyses, with a significance level of p < 0.05. The hierarchical regression model was built in stages, with variables entered in two blocks based on theoretical relevance: Yard descriptor variables were entered as block 1, as their impact on the spatial restrictions of the yard should be controlled for prior to investigating the impact of LFs on PPC. Variables encompassing two dimensional LFs were included in block 2. The fit of the model at each stage was compared using R-squared and adjusted R-squared, with a focus on the explanatory power of the model in relation to the number of variables. The model fit was evaluated by examining the residuals, estimating variance inflation factors (VIFs) to check for multicollinearity. The final model was chosen based on its ability to provide a meaningful explanation of the predictive ability of two dimensional LFs on PPC, while controlling for yard descriptors.

Semi-partial correlation coefficients squared were calculated to determine the percentage of variability of PPC uniquely accounted for by each predictor variable. Statistical significance was set at p<0.05.

## Results

**Fig 1:**
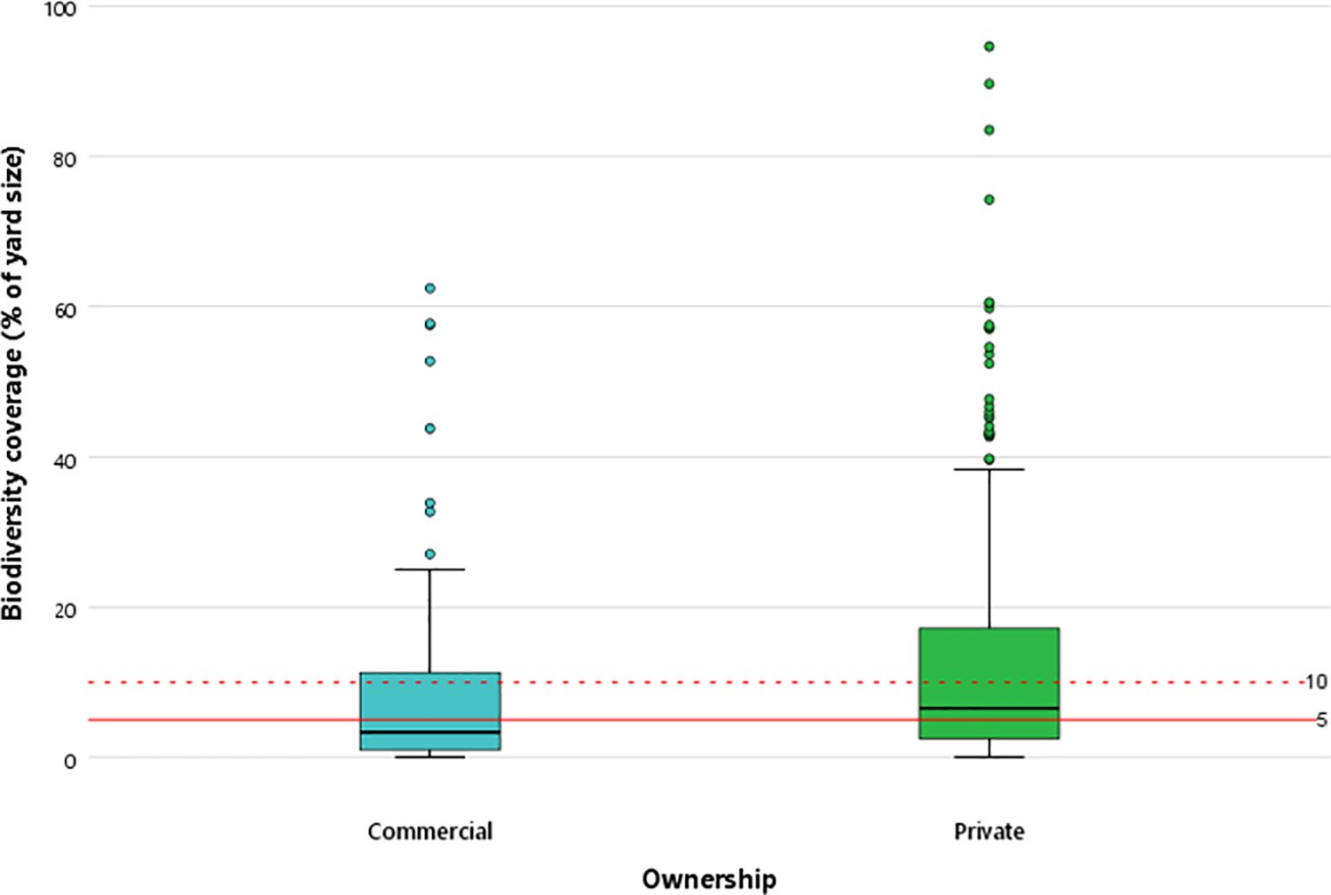
Percentage Property Coverage (PPC) of commercial vs. private yards.

**Fig 2:**
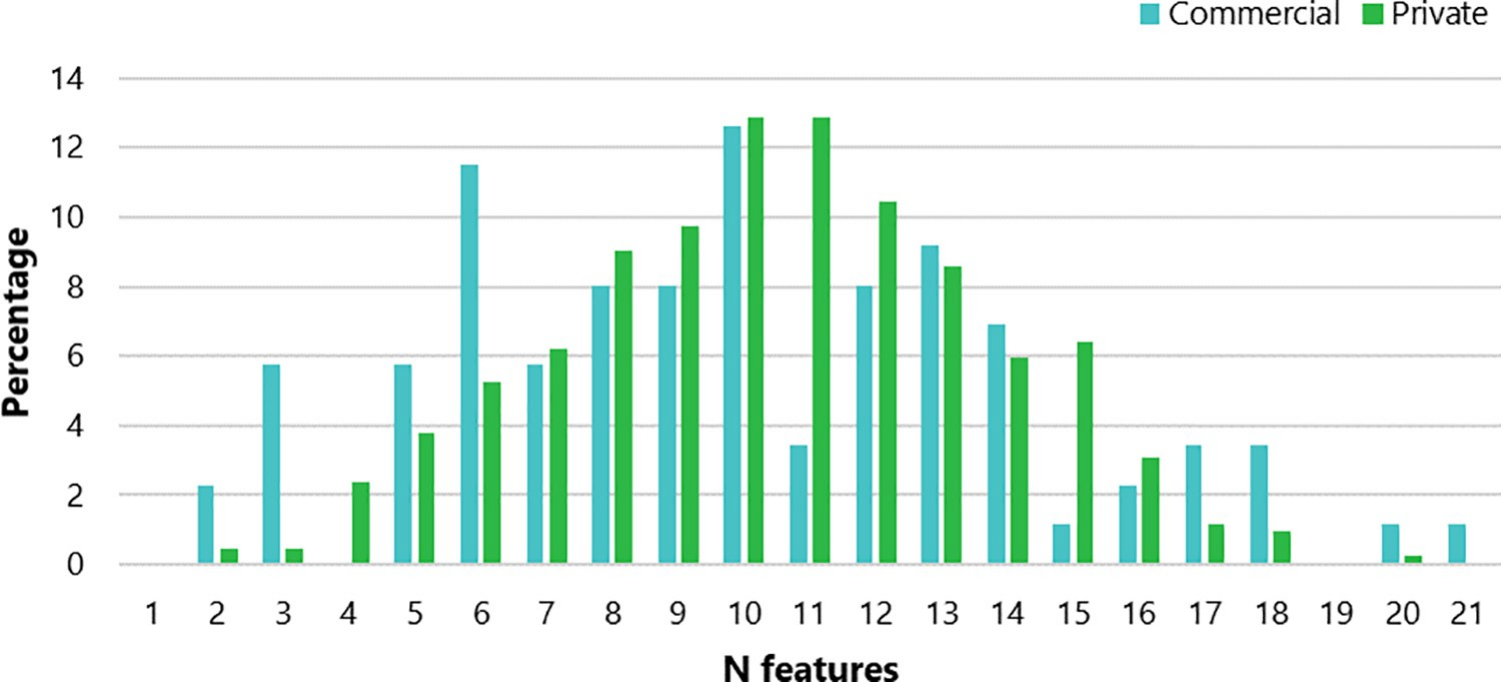
Percentage of commercial and private yards featuring an N number of LFs.

Commercial yards were significantly larger than private yards (p<0.001) and also had significantly more horses (p<0.001; Table 3). Commercial and private yards had a similar number of LFs but PPC was significantly higher on private yards (p<0.001; see Fig 1 and Table 3). All yards contained at least two features with only commercial yards featuring all 21 features (Fig 2). Most yards were situated on sandy soil, followed by clay soil.

**Table 3:** Descriptives of participating yards, including Mann-Whitney U test of differences.

	Overall	Commercial	Private	Differences between Commercial and Private yards (U-value; p-value)
**Participating yards (N) **	507	87 (17.2%)	420 (82.8%)	
**Yard size (ha)**				
Mean±SD	3.15±6.98	9.16±12.89***	1.9±3.97***	U = 6199;
Median; range	1.5;63.52	3.36;59.65	1.2; 63.52	<0.001
**Number of horses**				
Mean±SD	7.16±12.68	24.99±23.38***	3.47±1.54***	U = 1770.5;
Median; range	4;120	16; 120	3; 8	<0.001
**Number of LFs**				
Mean±SD	10.36±3.44	9.95±4.31	10.45±3.23	U = 16523;
Median; range	10;19	10;19	10; 18	0.159
**Percentage property coverage (PPC) in % with LFs**				
Mean±SD	11.77±14.66	9.08±13.87***	12.33±14.77***	U = 14163;
Median; range	5.85; 94.6	3.35; 62.43	6.57; 94.6	<0.001
**Type of soil**				
Clay	23.9%	29.9%	22.6%	N/a
Loam	3.6%	6.9%	2.9%	
Peat on sand	0.8%	2.3%	0.5%	
Peat	5.9%	3.4%	6.4%	
Sand	61.1%	49.4%	63.6%	
Silt loam	1.0%	2.3%	0.7%	
Don’t know	3.7%	5.7%	3.3%	

*<0.05

**<0.01

***<0.001

**Fig 3:**
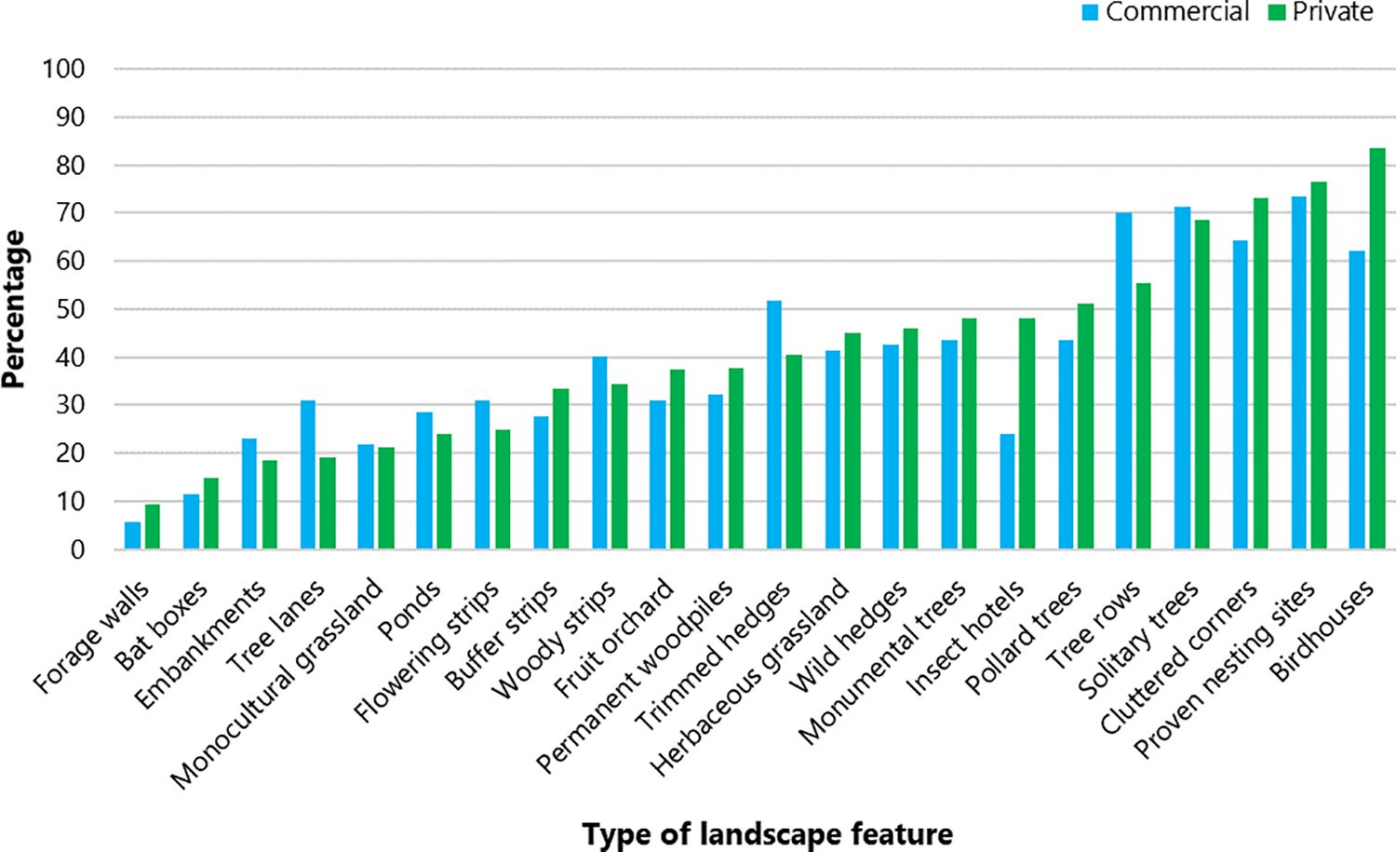
Proportion of commercial and private yards (in %) featuring individual LFs.

Most private and commercial yards contained LFs such as Birdhouses, Nesting sites and Cluttered corners (Fig 3). The least common LF was forage wall followed by bat boxes and embankments (Fig 3). In general, the proportion of yards containing a particular feature was comparable between private and commercial yards, with the most noticeable differences being features such as tree lanes, trimmed hedges and tree rows (Fig 3). Detailed descriptives of LFs for all yards is provided in the S1–S3 Tables.

### Univariable linear regression results

Fourteen of the 21 considered predictor variables in the univariate linear regression analyses explained significant variation in the percentage of a property covered by landscape features (Table 4). Yards that were larger and had more horses, had a lower PPC (Table 4). As can be expected, yards with more LFs also had a greater PPC on their property. Interestingly the size of monocultural grasslands did not explain PPC whilst the size of herbaceous grasslands did (Table 4). The variables explaining the most variation (as indicated through R-square) in PPC included Number of LFs (18.1%), Tree rows (8.4%) and Wild hedges (7.2%; Table 4). All other variables explained less than 5% of variation in PPC (Table 4).

**Table 4:** Model summary of univariable hierarchical linear regression.

Predictor variable	Unstandardized coefficients		Beta regression coefficients	t	Sig.	95% Confidence Interval		Semi partial correlation coefficients	R-square
	B	Std. Error				Lower Bound	Upper Bound		
Type of ownership	3.251	1.722	0.084	1.888	0.060	-0.132	6.634	0.084	0.007
Yard size (ha)	−0.344	0.092	−0.164	−3.739	<0.001	−0.525	−0.164	−0.566	0.027
Number of horses	-0.120	0.051	-0.104	-2.347	= 0.019	-0.221	-0.020	-0.104	0.011
Soil type	-0.204	0.303	-0.303	-0.675	= 0.500	-0.799	0.391	-0.030	0.001
Number of LFs	1.814	0.171	0.426	10.580	<0.001	1.477	2.151	0.426	0.181
Number of trees	0.043	0.034	0.095	1.278	= 0.203	-0.023	0.110	0.095	0.009
Tree lanes total m^2^	0.006	0.002	0.142	3.223	<0.001	0.002	0.009	0.142	0.020
Tree rows total m^2^	0.004	0.001	0.289	6.796	<0.001	0.003	0.005	0.289	0.084
Fruit orchard total m^2^	0.013	0.004	0.150	3.402	<0.001	0.005	0.020	0.150	0.022
Wild Hedges total m^2^	0.007	0.001	0.269	6.272	<0.001	0.005	0.009	0.269	0.072
Trimmed Hedges total m^2^	0.001	0.001	0.070	1.583	= 0.114	0.000	0.002	0.070	0.005
Monocultural grassland total m^2^	0.000	0.001	-0.033	-0.736	= 0.462	-0.002	0.001	-0.033	0.001
Herbaceous grassland total m^2^	0.001	0.000	0.118	2.660	0.008	0.000	0.002	0.118	0.014
Flowering strips total m^2^	0.005	0.001	0.244	5.664	<0.001	0.003	0.007	0.244	0.060
Woody strips total m^2^	0.003	0.001	0.196	4.485	<0.001	0.002	0.004	0.196	0.038
Forage walls total m^2^	-0.015	0.009	0.073	1.639	= 0.102	-0.003	0.033	0.073	0.005
Buffer strips total m^2^	0.005	0.001	0.324	7.690	<0.001	0.004	0.007	0.324	0.105
Ponds total m^2^	0.001	0.001	0.041	0.915	= 0.360	-0.001	0.004	0.041	0.002
Embankments total m^2^	0.002	0.000	0.205	4.718	<0.001	0.001	0.003	0.205	0.042
Wood piles total m^2^	0.038	0.014	0.123	2.784	= 0.006	0.011	0.064	0.123	0.015
Cluttered corners total m^2^	0.011	0.003	0.207	4.473	<0.001	0.006	0.016	0.207	0.043

### Multivariable hierarchical regression results

Inspection of the data, using the scatterplot of the standardised residuals, showed that assumptions of normality, linearity, and homoscedasticity had not been violated. Residuals were approximately rectangular in distribution with most of the scores concentrated along the 0 point. Multicollinearity was assessed by examining Tolerance and Variance Inflation Factor (VIF). Tolerance values ranged from 0.775 to 0.997 and the VIF ranged from 1.003 to 1.290, indicating no issues with multicollinearity.

Multivariable hierarchical linear regression analysis revealed that model 1, which included the yard descriptors, explained 21% of the variance (F(3,447) = 39.709; p < 0.001), with Yard size and Number of LFs contributing significantly to the model (Tables 5 and 6). Model 2, which also included the 14 LF variables explained 47.2% of the variation in PPC (F(14,436) = 27.815; p < 0.001; Tables 5 and 6). The following LF variables contributed significantly to the final model: Tree rows, Fruit orchard, Wild hedges, Flowering strips, Buffer strips, Embankments, Cluttered corners (Table 6).

**Table 5:** Model summary of multivariable hierarchical linear regression results.

Model	R	R Square	Adjusted R Square	Std. Error of the Estimate	R Square Change	F Change	df1	df2	Sig. F Change
1	0.459	0.21	0.205	13.0664	0.21	39.709	3	447	<0.001
2	0.687	0.472	0.455	10.82127	0.261	19.611	11	436	<0.001

**Table 6:** Multivariable hierarchical linear regression results.

Model		Unstandardized Coefficients		Beta regression coefficients	t	Sig.	Part	Collinearity Statistics	
		B	Std. Error					Tolerance	VIF
**1 **	Yard size (ha)	-0.335	0.095	-0.160	-3.532	<0.001	-0.148	0.864	1.157
	Number of horses	-0.028	0.052	-0.025	-0.543	0.587	-0.023	0.862	1.160
	Number of LFs	1.817	0.179	0.426	10.131	<0.001	0.426	0.997	1.003
**2 **	Yard size	-0.505	0.083	-0.241	-6.086	<0.001	-0.212	0.775	1.290
	Number of horses	-0.088	0.045	-0.076	-1.955	0.051	-0.068	0.794	1.260
	Number of LFs	0.987	0.167	0.232	5.898	<0.001	0.205	0.785	1.274
	Tree lanes total m^2^	0.002	0.001	0.050	1.342	0.180	0.047	0.877	1.140
	Tree rows total m^2^	0.003	0.000	0.207	5.386	<0.001	0.187	0.824	1.213
	Fruit orchard total m^2^	0.006	0.003	0.073	1.992	0.047	0.069	0.908	1.102
	Wild Hedges total m^2^	0.003	0.001	0.122	3.089	0.002	0.108	0.780	1.282
	Flowering strips total m^2^	0.003	0.001	0.156	4.242	<0.001	0.148	0.901	1.110
	Woody strips total m^2^	0.001	0.001	0.047	1.211	0.227	0.042	0.788	1.269
	Herbaceous grassland total m^2^	0.001	0.000	0.061	1.711	0.088	0.060	0.966	1.036
	Buffer strips total m^2^	0.004	0.001	0.276	7.831	<0.001	0.273	0.973	1.028
	Embankments total m^2^	0.002	0.000	0.147	3.980	<0.001	0.139	0.889	1.125
	Wood piles total m^2^	0.017	0.011	0.055	1.544	0.123	0.054	0.961	1.040
	Cluttered corners total m^2^	0.009	0.002	0.166	4.676	<0.001	0.163	0.957	1.045

^a^Dependent Variable: PPC

## Discussion

As the need for action to halt biodiversity loss becomes ever more urgent, previously overlooked sectors such as equestrianism [20, 24], are needed to contribute towards the restoration of ecosystem services. Our results emphasise the contribution that equine yards make towards promoting biodiversity and restoring ecosystem functions with an average property coverage of 9.08% (commercial yards) and 12.33% (private yards) for two-dimensional landscape features. These values already exceed the targets originally set out by the European Commission [17], and do not even consider the presence of trees on properties. Equine yards also tend to have a diverse number of LFs, with on average ten different features present on both commercial and private yards. These findings demonstrate that there are yards which manage to integrate landscape features effectively on their land. Moreover, current results reveal the additional biodiversity potential that equine yards can attain. The median coverage of 5.85% reflect that half of the yards have room for growth in integrating more landscape features into equine yards.

Not surprisingly, commercial and private yards differ significantly with regards to size and number of horses kept, with commercial yards managing, on average, 5 times as much land as private owners. However, there are five times as many private yard owners, indicating these types of owners, too, have access to a considerable amount of extensively used land.

Moreover, there is great diversity in yard design, which is not surprising considering the wide variety of equestrian properties After all, the way in which horses are kept is likely to vary depending on the primary purpose of the equine yard, ranging from sport and recreational activities, to coaching and therapy, breeding and trade and, on occasion, for the production of meat or milk or as working animals [20, 36, 37]. Such differences in yard design may be one of the key drivers to promoting biodiversity as the number of LFs explains signification variation in PPC. In particular, smaller yards with a greater number of LFs have greater PPC and landscape complexity. At the same time, design parameters to enhance biodiversity likely require complex considerations such as taking the purpose of the property into account and balancing yard size and the number of LFs. It could be argued, therefore, that much of the diversity observed is likely the result of the constraints and priorities of a yard’s primary purpose [38]. In order to integrate ecosystem services and stimulate biodiversity in equine yards, particular attention should be paid to the interaction between land utility and ecological processes [13], as well as how management and husbandry techniques may impact the ecological development of the landscape [39–42].

As current findings show, once the descriptor variables of a yard are controlled for, it is the type of features that determines ultimate PPC and provides a good indication of the actual biodiversity potential of equine yards. Buffer strips, wild hedges, tree rows, flowering strips, embankments and cluttered corners are all features shown to explain variation in the proportion LF coverage. Linear landscape features such as tree rows, buffer strips, wild hedges and flowering strips are widely heralded for their potential to preserve or even restore habitat diversity [43]. Due to their diversity in form and function, LFs can serve as effective secondary habitats for various species [44], promoting biodiversity conservation in otherwise species-poor landscapes [45, 46] as well as serving as important landscape corridors. Hedges and trees have been shown to provide multiple benefits, such as air and water filtration, pest control, pollination support, aesthetic and health advantages, erosion reduction, and annual carbon sequestration [47–51]. Flowering strips have been shown to provide a host of ecosystem services, most notably providing shelter and nectar to pollinators [52, 53] stimulating natural pest control [54] and increasing the aesthetic value of the environment [53, 55]. Equally, embankments, often viewed as engineered structures merely designed for flood control and water management, have become increasingly important as unique habitats to foster plant diversity, and enhance ecosystem functions [56, 57]. Current findings provide evidence that between one-third and one-half of equine yards already integrate a number of these key linear landscape features that, in equine yards at least, appear to be indicative of a more heterogenous yard design.

In the analysis, cluttered corners explained significant variation in PPC demonstrating their significant presence and contribution to landscape diversity. While cluttered corners are often negatively associated with, as the name would suggest, the cluttering of local landscapes and may also pose a certain safety risk for horses if not managed appropriately, they could potentially be modified into useful spaces. Such efforts may promote a change in perception of cluttered corners and in association with the diverse LF, contribute towards a perceptual shift regarding the role of the equine sector in the agro-ecological transition [20, 58].

The majority of equine yards are situated in peri-urban or rural settings, meaning that these yards may well be considered biodiversity ‘connecting hubs’. By linking other rural/agricultural properties with natural reserves, equine yards would facilitate interspecies exchange through landscape corridors [45] in areas where urbanisation or agricultural intensification would make it otherwise more difficult. What is more, since equine yards are not merely a source for horse-human interaction, but also for the interaction between humans and nature [39, 59], a more biodiverse-inclusive yard design may also allow visitors to the yard direct access to nature in the course of their interactions with horses.

Therefore, in order to engage a greater number of yard owners in more biodiverse yard design, cohesive strategies need to be employed on how to achieve such a shift. However, one-size-fits-all approaches to encourage the implementation of agri-ecological schemes have largely been found ineffective [60, 61], simply because they generally fail to take the specifics of a farm into account. In an industry as varied as equestrianism, it is important to take the experiences of other sectors on board.

Specifics of equine yard design are likely to be influenced by a number of factors, ranging from current levels of knowledge and skill, to personal preferences, ideology, client demands, financial considerations, ease of access and efficiency, and, importantly, aspects of equine health and welfare [27, 42, 62, 63].

Additional research is therefore required to investigate how equine yards could make full use of small landscape features while also impacting positively on different aspects of equine behaviour and welfare. In the context of sustainable business operations, it will also be essential to consider the diversity in the "biodiversity value" of different landscape features on the one hand and the efforts regarding maintenance on the other when developing new landscape designs. Cost-benefit analyses will need to determine how yard owners can best balance biodiversity value, equine health and welfare and business costs in light of geographic restrictions.

## Limitations

While this citizen science study offers valuable insights into the presence and quality of small landscape features (LFs) on equine yards, some limitations should be taken into account. As data collection relies on voluntary participation, equine yard owners may be more likely to have an interest in or knowledge about aspects relating to biodiversity, potentially leading to an overrepresentation of certain types of properties or specific practices [64]. This bias could affect the generalizability of the findings to all equine properties. For example, results from the study may potentially overestimate the PPC on equine yards, and the actual percentage may thus be lower. This, however, only emphasizes what can be achieved, with these yards acting as model yards that others can follow.

What is more, participants may have varying levels of expertise and commitment, leading to potential variations in data quality and consistency [65]. As a result, differences in data collection techniques, accuracy, and reporting standards can introduce noise into the dataset. However, some studies have shown that citizen scientists may, in fact, produce highly accurate results either due to awareness of their own novice status [66] or due to personal levels of competence [67].

The current study’s focus on equine properties in the Netherlands may limit the generalizability of the findings to other regions with different environmental conditions, land use patterns, and equine husbandry practices. Another variable that was also not measured in this study is how PPC may be influenced by the purpose of a yard, for example riding school, training stables or stud farms. Future research should investigate how the PPC of LFs varies among yard types and also among different countries and to understand how levels of biodiversity may be related to the PPC and types of LFs on a property. Despite these limitations, a citizen science approach can provide a valuable avenue for gathering data on LFs in equine properties, offering insights that may not be obtainable through traditional research methods. Careful consideration of these limitations is essential when interpreting and applying the study’s findings to broader contexts and policy recommendations.

## Conclusion

The current paper highlights the potential for the equine industry to contribute to the restoration of ecosystem services and the preservation of biodiversity through the integration of landscape features. However, despite encouraging results for the PPC of LFs on equine properties, the considerable variation between yards indicates that much remains to be done to integrate principles of biodiversity in equine yard design.

Key factors influencing landscape PPC included yard size, the number of landscape features, and the type of features. Smaller yards with more features had higher PPC and landscape heterogeneity, which likely enhance biodiversity. Linear landscape features such as tree rows, buffer strips, wild hedges, and flowering strips explained variation in the level of landscape feature coverage.

To encourage more biodiversity on equestrian properties, tailored strategies should consider the diverse factors influencing equine yard design, including existing knowledge, client demands, financial considerations, and equine health and welfare.

## Supporting information

S1 Table Descriptives of landscape features overall yards. (DOCX)

S2 Table Descriptives of landscape features: Commercial yards. (DOCX)

S3 Table Descriptives of landscape features: Private yards. (DOCX)
