# Supplementary material for: Green treasures: Investigating the biodiversity potential of equine yards through the presence and quality of landscape features in the Netherlands
Source: PLoS One. 2024 Apr 11;19(4):e0301168. doi: 10.1371/journal.pone.0301168 (PMC11008862; doi:10.1371/journal.pone.0301168)
Supplement: S1 Table — (DOCX) [file pone.0301168.s001.docx]

**S1 Table: Descriptives of Landscape features overall yards (^a^ = circumference; ^b^ = m^2^)**

|  | **Overall** | | | | | | | | |
| --- | --- | --- | --- | --- | --- | --- | --- | --- | --- |
| **Type of landscape and biodiversity features** | **% of yards** | **No. (Mean±SD)** | **No. (Median; range)** | **Length (Mean±SD)** | **Length (Median; range)** | **Width (Mean±SD)** | **Width (Median; range)** | **Total average size of one LF (Mean±SD)** | **Total average size of one LF (Median;range)** |
| **Solitary tree** | 69 | 7.62±10.27 | 5.00;100 | n/a | n/a | n/a | n/a | 73.99±64.19 **^a^** | 50;400 **^a^** |
| **Monumental tree** | 47,3 | 5.08±7.65 | 3.00;75 | n/a | n/a | n/a | n/a | 125.23±82.12 **^a^** | 100;600 **^a^** |
| **Pollard tree** | 49,9 | 15.59±27.31 | 9.00;350 | n/a | n/a | n/a | n/a | 54.71±47.94 **^a^** | 40;300 **^a^** |
| **Tree lane** | 21,1 | 1.19±0.82 | 1.00;6 | 91.4±110.92 | 50;604 | 3.91±2.28 | 4.00;12 | 439.28±543.83 | 280;3500 |
| **Tree row** | 58 | 2.33±1.96 | 2.00;13 | 82.67±92.82 | 56.25;797 | 3.61±2.32 | 3.00;14 | 301.53±426.51 | 180;3997 |
| **Fruit orchard** | 36,3 | 1±0.00 | 1;0 | 18.58±20.72 | 10;149.5 | 7.69±6.35 | 5.00;19 | 170.50±248.65 | 60;1599 |
| **Wild hedge** | 45,4 | 1.95±1.21 | 2.00;7 | 72.55±92.84 | 50;595 | 2.79±1.93 | 2.00;14 | 200.38±302.80 | 100;2495 |
| **Trimmed hedge** | 42,4 | 3.16±3.21 | 3.00;29 | 80.83±111.38 | 44;996 | 1.58±1.06 | 1.00;5 | 125.29±197.21 | 60;1596 |
| **Woody strip** | 35,3 | 1.65±0.95 | 1.00;4 | 91.89±125.34 | 50;798 | 4.17±3.24 | 3.00;19 | 405.62±645.01 | 200;3997 |
| **Forage wall** | 8,9 | 1.91±1.40 | 1.00;6 | 40.43±45.86 | 25;247.5 | 2.00±1.12 | 2.00;4 | 72.44±75.48 | 60;387 |
| **Flowering strip** | 26 | 2.01±1.96 | 1.00;19 | 57.10±102.90 | 20;697 | 2.67±2.58 | 2.00;19 | 181.23±387.34 | 40;1997 |
| **Embankment** | 19,3 | 2.05±1.53 | 1.00;7 | 195.76±339.39 | 70;2498 | 3.44±3.27 | 3.00;19 | 426.46±670.71 | 225;3746 |
| **Buffer strip** | 32,5 | 1.93±1.38 | 2.00;9 | n/a | n/a | n/a | n/a | 284.19±550.82 | 100;4999 |
| **Monocultural grassland** | 21,5 | n/a | n/a | n/a | n/a | n/a | n/a | 982.49±1599.66 | 1.20;5000 |
| **Herbaceous grassland** | 44,4 | n/a | n/a | n/a | n/a | n/a | n/a | 995.21±1527.50 | 25;5000 |
| **Pond** | 24,9 | 1.14±0.41 | 1.00;3 | n/a | n/a | n/a | n/a | 305.83±638.29 | 78.54;4417.08 |
| **Permanent woodpile** | 36,9 | 1.69±1.32 | 1.00;9 | n/a | n/a | n/a | n/a | 15.27±73.85 | 5.00;999.50 |
| **Cluttered corner** | 71,8 | 2.45±3.16 | 2.00;39 | n/a | n/a | n/a | n/a | 37.58±220.86 | 8.50;3999.50 |
| **Birdhouse** | 79,9 | 6.78±5.80 | 5.00;37 | n/a | n/a | n/a | n/a | n/a | n/a |
| **Bat box** | 14,2 | 1.69±1.03 | 1.00;4 | n/a | n/a | n/a | n/a | n/a | n/a |
| **Insect hotel** | 44,2 | 2.23±1.85 | 2.00;12 | n/a | n/a | n/a | n/a | n/a | n/a |
| **Proven nesting site** | 76,1 | 7.95±17.10 | 4.00;250 | n/a | n/a | n/a | n/a | 15.93±43.70 | 2.00;800 |
