# Supplementary material for: Green treasures: Investigating the biodiversity potential of equine yards through the presence and quality of landscape features in the Netherlands
Source: PLoS One. 2024 Apr 11;19(4):e0301168. doi: 10.1371/journal.pone.0301168 (PMC11008862; doi:10.1371/journal.pone.0301168)
Supplement: S2 Table — (DOCX) [file pone.0301168.s002.docx]

**S2 Table:** **Descriptives of Landscape features: commercial yards (^a^ = circumference; ^b^ = m^2^)**

|  | **Commercial** | | | | | | | | |  | |
| --- | --- | --- | --- | --- | --- | --- | --- | --- | --- | --- | --- |
| **Type of landscape and biodiversity features** | **% of yards** | **No. (Mean±SD)** | **No. (Median; range)** | **Length (Mean±SD)** | **Length (Median;range)** | **Width (Mean±SD)** | **Width (Median;range)** | **Total average size of one LF (Mean±SD)** | **Total average size of one LF (Median; range)** | |  |
| **Solitary tree** | 71,3 | 12.47±17.77 | 5.00;99 | n/a | n/a | n/a | n/a | 76.50±52.25 **^a^** | 60;240 **^a^** | |  |
| **Monumental tree** | 43,7 | 5.79±7.71 | 3.00;39 | n/a | n/a | n/a | n/a | 121.39±104.87 **^a^** | 100;599 **^a^** | |  |
| **Pollard tree** | 43,7 | 23.87±56.84 | 8.50;349 | n/a | n/a | n/a | n/a | 73.55±43.96 **^a^** | 70;190 **^a^** | |  |
| **Tree lane** | 31 | 1.27±1.14 | 1.00;6 | 125.55±142.78 | 70;500 | 3.72±2.20 | 4.00;10 | 619.57±829.33 **^b^** | 300;3500 | |  |
| **Tree row** | 70,1 | 3.33±2.75 | 3.00;12 | 116.85±128.76 | 80;790 | 3.42±2.48 | 3.00;14 | 385.09±583.11 **^b^** | 200;3990 | |  |
| **Fruit orchard** | 31 | 1±0.00 | 1;0 | 18.87±20.68 | 10;88 | 7.03±6.31 | 5.00;19 | 157.04±242.29 | 45;798 | |  |
| **Wild hedge** | 42,5 | 2.24±1.48 | 2.00;7 | 72.16±98.60 | 40;495 | 3.46±3.15 | 3.00;14 | 213.78±286.61 | 100;1190 | |  |
| **Trimmed hedge** | 51,7 | 3.71±4.50 | 3.00;29 | 111.63±191.23 | 45;992.5 | 2.38±1.67 | 2.00;4 | 200.33±293.82 | 130;1592.5 | |  |
| **Woody strip** | 40,2 | 1.71±0.99 | 1.00;4 | 130.64±184.45 | 50;797.6 | 4.33±3.04 | 4.00;9 | 624.54±1033.70 | 240;3995 | |  |
| **Forage wall** | 5,7 | 1.60±1.34 | 1.00;3 | 41.00±20.74 | 40;55 | 2.90±1.34 | 3.00;3.5 | 113.00±67.79 | 100;165 | |  |
| **Flowering strip** | 31 | 2.63±3.63 | 2.00;19 | 91.26±160.62 | 25;697 | 3.02±2.26 | 2.50;11 | 310.48±575.31 | 60;1997 | |  |
| **Embankment** | 23 | 2.65±1.66 | 2.00;6 | 282.00±315.05 | 150;995 | 3.66±3.04 | 3.00;14 | 923.00±1048.59 | 325;3725 | |  |
| **Buffer strip** | 27,6 | 2.42±1.89 | 2.00;9 | n/a | n/a | n/a | n/a | 266.75±367.57 | 112.5;1398 | |  |
| **Monocultural grassland** | 21,8 | n/a | n/a | n/a | n/a | n/a | n/a | 469.17±1196.56 | 0.00;5000 | |  |
| **Herbaceous grassland** | 41,4 | n/a | n/a | n/a | n/a | n/a | n/a | 620.27±1130.98 | 55;5000 | |  |
| **Pond** | 28,7 | 1.24±0.44 | 1.00;1 | n/a | n/a | n/a | n/a | 558.29±978.46 | 176.71;3847.67 | |  |
| **Permanent woodpile** | 32,2 | 1.82±1.28 | 1.00;5 | n/a | n/a | n/a | n/a | 10.18±14.79 | 5.00;73 | |  |
| **Cluttered corner** | 64,4 | 2.84±2.87 | 2.00;17 | n/a | n/a | n/a | n/a | 25.43±36.26 | 10;199 | |  |
| **Birdhouse** | 62,1 | 6.07±4.84 | 5.00;24 | n/a | n/a | n/a | n/a | n/a | n/a | |  |
| **Bat box** | 11,5 | 1.50±0.97 | 1.00;3 | n/a | n/a | n/a | n/a | n/a | n/a | |  |
| **Insect hotel** | 24,1 | 2.48±2.25 | 2.00;9 | n/a | n/a | n/a | n/a | n/a | n/a | |  |
| **Proven nesting site** | 73,6 | 11.80±16.50 | 5.00;99 | n/a | n/a | n/a | n/a | 29.36±99.76 | 10;800 | |  |
