# Supplementary material for: Green treasures: Investigating the biodiversity potential of equine yards through the presence and quality of landscape features in the Netherlands
Source: PLoS One. 2024 Apr 11;19(4):e0301168. doi: 10.1371/journal.pone.0301168 (PMC11008862; doi:10.1371/journal.pone.0301168)
Supplement: S3 Table — (DOCX) [file pone.0301168.s003.docx]

**S3 Table: Descriptives of Landscape features: private yards (^a^ = circumference; ^b^ = m^2^)**

|  | **Private** | | | | | | | | |
| --- | --- | --- | --- | --- | --- | --- | --- | --- | --- |
| **Type of landscape and biodiversity features** | **% of yards** | **No. (Mean±SD)** | **No. (Median; range)** | **Length (Mean±SD)** | **Length (Median;range)** | **Width (Mean±SD)** | **Width (Median;range)** | **Total average size of one LF (Mean±SD)** | **Total average size of one LF (Median; range)** |
| **Solitary tree** | 68,6 | 6.6±7.48 | 4.00;64 | n/a | n/a | n/a | n/a | 73.46±66.50 **^a^** | 50;400 **^a^** |
| **Monumental tree** | 48,1 | 4.95±7.66 | 3.00;75 | n/a | n/a | n/a | n/a | 125.95±77.45 **^a^** | 100;400 **^a^** |
| **Pollard tree** | 51,2 | 14.13±17.53 | 9.50;100 | n/a | n/a | n/a | n/a | 51.40±47.94 **^a^** | 40;300 **^a^** |
| **Tree lane** | 19 | 1.17±0.68 | 1.00;4 | 79.76±95.97 | 50;604 | 3.97±2.32 | 4.00;12 | 376.96±389.64 **^b^** | 280;2416 |
| **Tree row** | 55,5 | 2.07±1.61 | 1.00;13 | 73.72±78.78 | 50;697 | 3.66±2.28 | 3.00;9 | 279.66±373.48 **^b^** | 160;3997 |
| **Fruit orchard** | 37,4 | 1±0.00 | 1;0 | 18.53±20.80 | 10;149.5 | 7.81±6.38 | 5.00;19 | 172.81±250.41 **^b^** | 60;1599 |
| **Wild hedge** | 46 | 1.89±1.15 | 2.00;7 | 72.62±91.97 | 50;595 | 2.66±1.58 | 2.00;9 | 197.81±306.46 **^b^** | 100;2495 |
| **Trimmed hedge** | 40,5 | 3.01±2.78 | 2.00;28 | 72.68±76.61 | 42;396 | 1.37±0.70 | 1.00;5 | 105.42±157.71 **^b^** | 50;1596 |
| **Woody strip** | 34,3 | 1.64±0.94 | 1.00;4 | 82.47±104.92 | 50;698 | 4.14±3.30 | 3.00;19 | 352.41±499.22 | 170;3497 |
| **Forage wall** | 9,5 | 1.95±1.41 | 1.00;6 | 40.36±48.25 | 20;247.5 | 1.89±1.06 | 1.50;4 | 67.37±75.62 | 42.50;387 |
| **Flowering strip** | 25 | 1.85±1.18 | 1.00;5 | 48.32±80.67 | 20;597 | 2.58±2.65 | 2.00;19 | 148.00±317.47 | 35;1977 |
| **Embankment** | 18,6 | 1.90±1.46 | 1.00;7 | 173.64±343.78 | 60;2498 | 3.38±3.34 | 3.00;19 | 344.38±475.58 | 190;2496 |
| **Buffer strip** | 33,6 | 1.85±1.26 | 1.00;6 | n/a | n/a | n/a | n/a | 287.16±577.20 | 100;4999 |
| **Monocultural grassland** | 21,4 | n/a | n/a | n/a | n/a | n/a | n/a | 1161.80±1685.28 | 2.30;5000 |
| **Herbaceous grassland** | 45 | n/a | n/a | n/a | n/a | n/a | n/a | 1064.50±1581.80 | 15;5000 |
| **Pond** | 24 | 1.12±0.41 | 1.00;3 | n/a | n/a | n/a | n/a | 243.34±509.53 | 78.54;4414.72 |
| **Permanent woodpile** | 37,9 | 1.67±1.33 | 1.00;9 | n/a | n/a | n/a | n/a | 16.17±79.86 | 5.00;999.50 |
| **Cluttered corner** | 73,3 | 2.38±3.21 | 2.00;39 | n/a | n/a | n/a | n/a | 39.79±239.61 | 8.00;3999.50 |
| **Birdhouse** | 83,6 | 6.89±5.93 | 5.00;37 | n/a | n/a | n/a | n/a | n/a | n/a |
| **Bat box** | 14,8 | 1.73±1.04 | 1.00;4 | n/a | n/a | n/a | n/a | n/a | n/a |
| **Insect hotel** | 48,3 | 2.20±1.81 | 2.00;12 | n/a | n/a | n/a | n/a | n/a | n/a |
| **Proven nesting site** | 76,7 | 7.18±17.14 | 3.00;250 | n/a | n/a | n/a | n/a | 13.25±17.00 | 2.00;40 |
